# A lightning cluster identification method considering multi-scale spatiotemporal neighborhood relationships

**DOI:** 10.1371/journal.pone.0333207

**Published:** 2025-10-03

**Authors:** Manxing Shi, Peng Fan, Hantao Tao, Qin Li, Ju Wang, Yujun Liu, Lai Wei

**Affiliations:** 1 School of Geographic Sciences, Xinyang Normal University, Xinyang, China; 2 School of New Energy and Electrical Engineering, Hubei University, Wuhan, China; 3 China Electric Power Research Institute, Wuhan, China; 4 Spatial Information Technology Application Department, Changjiang River Scientific Research Institute, Wuhan, China; ICIMOD: International Centre for Integrated Mountain Development, NEPAL

## Abstract

Rapid and accurate identification and tracking of lightning clusters from massive lightning detection data are crucial for real-time thunderstorm nowcasting and climatological analyses of thunderstorm activity. Although density-based clustering algorithms can identify clusters of arbitrary shapes at fine scales, their performance is often hindered by large data volumes and significant variations in lightning density. To address these challenges, we propose a multi-scale spatiotemporal lightning clustering framework, termed CC3D-CSCAP. It consists of two main components. First, the 3-D connected component algorithm (CC3D) performs coarse-scale segmentation by dividing the lightning dataset into spatiotemporally disconnected subsets using 26-connectivity. Then, the cylinder-based scan clustering algorithm with adaptive parameters (CSCAP) is applied to each subset for fine-scale identification of lightning clusters. Since the lightning subset may still contain multiple thunderstorms with varying lightning densities, CSCAP adaptively determines clustering parameters based on the statistical characteristics (time difference and spatial distance) of subset. Compared with fixed-parameter methods, CC3D-CSCAP identifies more clusters (771,033) while retaining a high percentage of usable lightning strokes (98.988%). The clustering results align well with the theoretical criteria for optimal clustering and are promising for global applications in lightning data analysis, nowcasting, and climatological studies of convective systems.

## Introduction

A thunderstorm, often referred to as an electrical storm, is a localized convective system that consistently develops from cumulonimbus clouds and is invariably associated with lightning and thunder [1]. Intense thunderstorms are typically accompanied by severe weather phenomena, including tornadoes, hail, damaging convective winds, and short-duration heavy precipitation [2–4]. Some studies [5,6] suggest that lightning detection data serve as one of the most objective samples representing thunderstorm activity. However, lightning sensors typically provide only the time and location of lightning data, lacking a clear definition of thunderstorm events [7,8]. Consequently, the development of efficient and robust methods for identifying and extracting thunderstorm activity from massive lightning datasets has become an important research topic.

Scientists use the term lightning stroke to describe the rapid discharge of electric charge between a cloud and the ground, which may occur multiple times within a single lightning flash [9–11]. Some lightning strikes observed by the Beijing Lightning Network (BLNET) were located with more than 400 associated lightning pulses [12]. Initially, many researchers focused on the triggering of individual lightning strokes without considering the characteristics and properties of successive lightning sequences. This was primarily due to the assumption that lightning is a discrete and random process, with each event occurring independently and without relation to others [13]. Yair et al. [14] applied the Kolmogorov–Smirnov (KS) test to examine whether the distribution of inter-stroke intervals follows an exponential distribution and found that lightning stroke sequences within isolated thunderstorm cells or multi-cell thunderstorm systems are not random. The co-occurrence of lightning and thunderstorms reveals a vital pattern: all lightning strokes generated during a thunderstorm event inevitably occur within a specific spatial coverage and duration, reflecting the inherent physical characteristics of the thunderstorm system.

The identification and tracking of lightning clusters from lightning detection data have garnered substantial attention from researchers worldwide [5]. To support various research objectives, a range of clustering techniques have been proposed and refined, aiming to improve thunderstorm forecasting and enhance understanding of storm dynamics [8,15–18]. In recent years, both grid-based and density-based clustering techniques have been widely applied in the study of thunderstorm identification.

The grid-based approach partitions the observation domain into regular grid cells [19–21], assigns lightning events to corresponding cells, and subsequently performs clustering analysis on the grid level to delineate thunderstorm systems. Huang et al. [22] applied the eight-connected component labeling method to total lightning data observed by the Foshan Lightning Location System to investigate how varying spatiotemporal parameters influence thunderstorm identification. Their results showed that using grid sizes of 0.02° to 0.05° yielded cluster areas that were approximately 4, 9, 16, and 25 times larger than those identified with a 0.01° grid. Mezuman et al. [23] and Harel and Price [15] transformed lightning observations from the Worldwide Lightning Location Network (WWLLN) into density matrices with a horizontal resolution of 0.15° and a temporal resolution of 1 hour. During the binarization process, grid cells containing at least one lightning stroke were assigned a value of 1, while all others were set to 0. Connected component analysis was then applied to identify spatial clusters, enabling the investigation of thunderstorm activity on both global and African scales. Grid-based clustering methods offer several advantages: (1) they are conceptually simple and easy to implement without requiring complex mathematical models; (2) they are computationally efficient, as the transformation of discrete lightning strokes into density fields reduces processing complexity; and (3) they are highly scalable, with adjustable spatial and temporal resolutions that allow for application to large datasets.

Density-based clustering algorithms aim to identify high-density regions within unlabeled datasets [24,25]. A cluster is typically defined as a group of points that converge toward the same local maximum in the density distribution. Points within a cluster are closely spaced while being relatively distant from points in other dense regions [26]. Hutchins et al. [8] extended the density-based spatial clustering of applications with noise (DBSCAN) algorithm into the spatiotemporal domain. Sensitivity tests demonstrated that the optimal clustering parameters for WWLLN data are a maximum spatial radius of 0.12°, a maximum temporal window of 18 minutes, and a minimum density threshold of two strokes. Galanaki et al. [7] applied a cylindrical spatiotemporal scanning method to analyze Mediterranean thunderstorm climatology from 2005 to 2014. They found that a spatial threshold of 0.10° and a temporal window of 16 minutes yielded the most climatologically meaningful clustering results. This method employs a three-dimensional cylindrical scanning window, where the circular base represents spatial proximity and the height corresponds to temporal proximity [27–30]. Density-based clustering algorithms exhibit several key characteristics: (1) robustness to outliers and noise; (2) no requirement to predefine the number of clusters; (3) the capability to identify clusters of arbitrary shapes; and (4) insensitivity to the temporal or spatial ordering of lightning data.

Unfortunately, both clustering approaches have inherent limitations due to the discrete nature and variable density of lightning data. Grid-based clustering is highly sensitive to grid resolution: overly fine grids may reduce classification accuracy, while coarse grids fail to capture small-scale thunderstorm cells [31]. Density-based clustering faces challenges such as high computational cost and sensitivity to parameter selection [22,32]. Small parameter values can result in an excessive number of clusters with a low proportion of meaningful lightning events, as multicell thunderstorms may fragment and sparse strokes may be excluded. Conversely, relaxed parameters preserve more lightning data but risk merging adjacent convective cells into a single cluster. Consequently, clustering algorithms with fixed parameters become less effective when the lightning density varies significantly across storm systems.

This paper proposes a lightning cluster identification method (S1 Fig) that incorporates multiscale spatiotemporal neighborhood relationships, effectively addressing the limitations of both grid-based and density-based approaches. The proposed method combines a three-dimensional connected component algorithm (CC3D) for coarse-scale identification with a cylinder scan clustering algorithm using adaptive parameters (CSCAP) for fine-scale detection. This hybrid approach reduces computational complexity while effectively capturing lightning clusters with variable-density lightning distributions. Lightning detection data are gridded using sufficiently large spatial and temporal intervals, and the CC3D algorithm is applied to track spatiotemporally continuous thunderstorm grid cells, assigning the same label to adjacent cells in space and time. Then, all grid cells sharing the same label are associated with lightning strokes occurring within their corresponding spatial and temporal ranges, forming a lightning data subset. Finally, each subset is subjected to fine-scale identification using the density-based CSCAP algorithm.

## Material

### Study area

**Fig 1:**
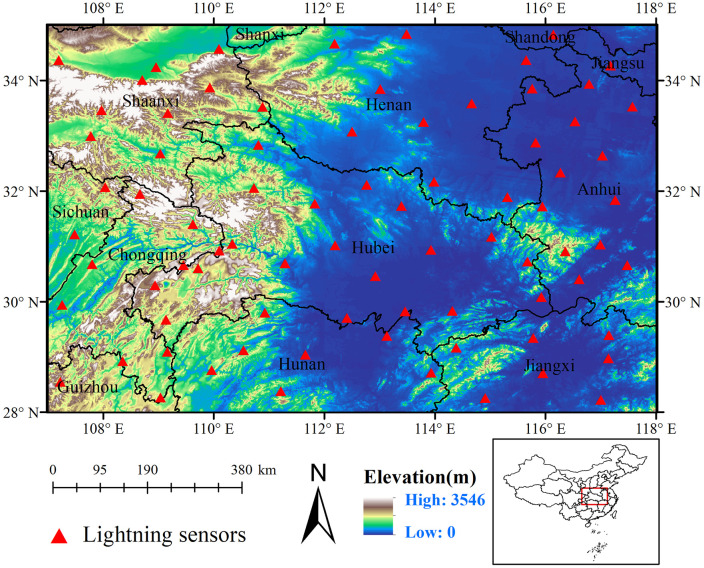
Topographic map of the Central China. The filled red triangles represent the lightning sensors within the domain. Elevation data obtained from NASA's Shuttle Radar Topography Mission (SRTM).

The study area, situated in Central China (28°N–35°N, 107°E–118°E), is located within a transitional zone of China's topographic staircases, characterized by diverse landforms that include mountains, hills, lakes, and plains (Fig 1). Terrain variations contribute to significant spatial heterogeneity in land cover. Although the region is inland, large rivers and lakes supply abundant moisture, facilitating convective development. Thunderstorm activity is widespread in the southeastern part during the warm season, driven by complex interactions between topography, moisture availability, and atmospheric dynamics [33].

### Data

The cloud-to-ground (CG) lightning data (S2 Data) analyzed in this study were collected from 2013 to 2015 using the Lightning Location System (LLS) developed and maintained by the State Grid Electric Power Research Institute [34]. A total of 80 lightning sensors are deployed across the study area, providing uniform spatial coverage with an average inter-sensor spacing of less than 300 km. LLS employs a hybrid positioning technique that integrates time-of-arrival (TOA) and magnetic direction methods to enhance detection accuracy [35]. Each lightning event is recorded with detailed attributes, including occurrence time, geographic coordinates, peak current magnitude, polarity, and the number of sensors involved in its detection [36]. To ensure data reliability, a rigorous quality control process is applied to eliminate misclassified cloud flashes. Firstly, return strokes with absolute peak currents below 10 kA are excluded, as they are more likely to originate from cloud lightning [37]. Secondly, lightning events detected by an insufficient number of sensors are removed to minimize localization uncertainty.

## Methodology

**Fig 2:**
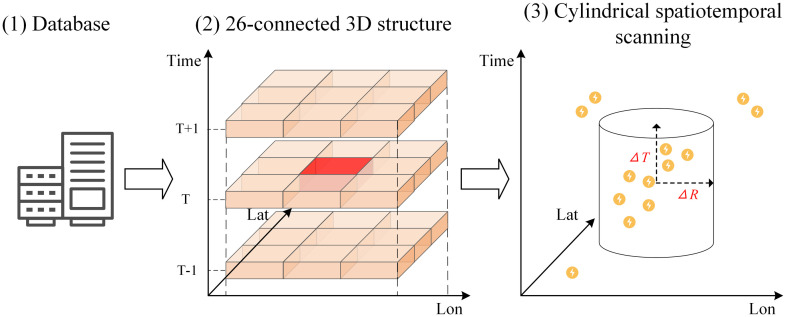
A multi-scale spatiotemporal neighborhood-based approach for identifying lightning clusters.

Although return strokes are distinct from lightning flashes, this study utilizes return stroke information to identify and extract thunderstorm activity [34]. The fundamental theory posits that all lightning discharges associated with a single thunderstorm event must occur within a defined spatial extent and temporal duration, reflecting the intrinsic physical characteristics of the parent convective system. We propose a multi-scale spatiotemporal clustering framework (Fig 2) for large-scale lightning stroke data (https://github.com/XYNU-MXS/CC3D-CSCAP.git). Lightning strokes are first mapped onto 3D binary grids, where 26-connected component labeling identifies temporally and spatially independent stroke subsets. A cylindrical scanning algorithm is then applied within each subset to extract multiple coexisting thunderstorm systems. By applying adaptive neighborhood thresholds to each subset, the method accommodates variations in lightning density while significantly reducing computational complexity. This approach effectively addresses challenges related to density heterogeneity and large data volumes, providing a scalable and structure-aware solution for thunderstorm identification based on lightning observations.

### Coarse-scale identification of grid clusters based on the grid-based CC3D algorithm

CC3D is a Python package (https://pypi.org/project/connected-components-3d/) developed initially by Silversmith for labeling three-dimensional biomedical imagery, particularly brain tissue datasets. The algorithm is a detailed extension of the two-dimensional connected components labeling (CCL) method initially developed by Rosenfeld and Pfaltz [38] and adapted for three-dimensional imagery. It utilizes an equivalence table to implement a union-find structure with path compression and balancing strategies. Moreover, the algorithm is enhanced by incorporating the decision tree proposed by Wu, Otoo, and Suzuki [39], which is commonly referred to as the scan plus array-based union-find (SAUF) method. In recent years, CC3D has demonstrated promising potential for identifying meteorological phenomena such as extreme heatwaves in climate studies [40,41].

A horizontal distance of 0.25° and a temporal interval of 1 hour are sufficient to distinguish lightning strokes associated with different thunderstorm systems. Empirically, the maximum spatial and temporal thresholds between two successive lightning strokes within a single thunderstorm are commonly set at approximately 10 kilometers and 15 minutes, respectively [7,8]. The gridded lightning data were converted into a binary image by applying a density threshold of one lightning event; grid cells meeting this criterion were assigned a value of 1, while cells with no lightning occurrences were set to 0. The CC3D algorithm employed in this study extends the traditional CCL method to 26-connected neighborhoods in three dimensions, enabling the identification of 26-connected components within 3D imagery. A three-dimensional binary image with dimensions of latitude (0.25°) × longitude (0.25°) × time (1 h) was input into the CC3D algorithm to identify all potentially connected thunderstorm grid cells.

The spatial displacement and temporal evolution of thunderstorms manifest as a 26-connected three-dimensional structure within a gridded spatiotemporal domain. Once each lightning record is linked to this structure, it acquires distinct physical properties. Several metrics, including frequency, maximum areal coverage, intensity, and spatial distribution, can characterize lightning-associated grid clusters. The centroid of each cluster is computed by applying a weighted average to the geographic coordinates of the grid cells, where the lightning counts determine the weights. Maximum areal coverage refers to the potential extent influenced by lightning activity, corresponding to the total number of grid cells within a single cluster. Cluster intensity is quantified by the number of lightning strokes per unit grid cell, defined as the ratio of total strokes to the number of cells in the cluster.

**Fig 3:**
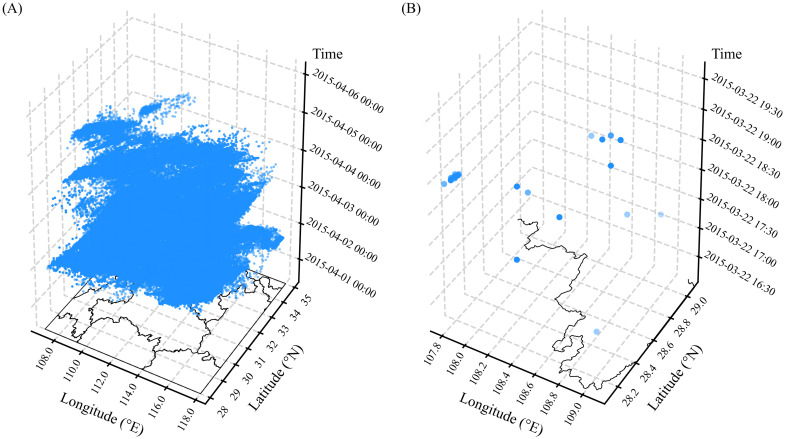
Two representative subsets of (A) high-flash-rate and (B) low-flash-rate lightning data identified by the CC3D algorithm.

The 26-connectivity refers to scanning from the center of a 3 × 3 × 3 cube (Fig 2 (2)), fully accounting for the spatial neighborhood at the current time as well as one hour before and after. The algorithm is capable of extracting all spatially and temporally adjacent lightning events into a single lightning data subset to the greatest extent possible. The relatively coarse spatiotemporal resolution ensures that different lightning data subsets are uncorrelated in space and time. However, an individual lightning data subset is not considered the final identification result. Although the high-flash-rate and low-flash-rate subsets exhibit considerable differences in density, both may encompass multiple thunderstorm systems (Fig 3). Therefore, it is necessary to further develop a fine-scale identification algorithm with adaptive clustering parameters for the lightning subsets derived from the CC3D algorithm.

### Fine-scale identification of lightning clusters based on the density-based CSCAP algorithm

#### Clustering principle based on cylindrical scanning.

The cylindrical scan clustering method shares a similar principle with ST-DBSCAN [42], using density as a metric to quantify the similarity between distinct events. The algorithm searches the feature space for high-density regions separated by areas of low density. A predefined threshold is applied to identify dense regions within which events are assigned to the same cluster. The threshold refers to the spatiotemporal neighborhood and the density parameter (*MinPts*) [7,8]. The algorithm typically employs a cylindrical window defined by a spatial radius (*ΔR*) and temporal interval (*2ΔT*) to effectively estimate the density around a central point (Fig 2 (3)). When the density exceeds *MinPts*, the point is identified as a core point. The spatiotemporal scanning-based clustering approach in this study treats each lightning event within a subset of lightning data (*D*) as a candidate core point. A cylindrical neighborhood is defined around each event to evaluate whether it qualifies as a core point.

Definition of the cylindrical neighborhood: Taking event *p* as the center, its neighborhood is the set of all events *q* within a three-dimensional spacetime with a radius of *ΔR* and a time interval of *ΔT*.


$$N_{neighborhood}(p)=\{q\in D\;|\;DistS(p,q)\leq \Delta R\;and\;DistT(p,q)\leq |\Delta T|\}$$ (1)

Here, *D* denotes a lightning data subset identified by the CC3D algorithm; *DistS*(*p*, *q*) represents the Euclidean distance between events *p* and *q*; and *DistT*(*p*, *q*) denotes the time difference between events *p* and *q*.

Definition of the core point: For the subset *D* of lightning data, with the density threshold *MinPts* set to 2, if event *p* in *D* satisfies Equation (2), then event *p* is considered a core point.


$$|N_{neighborhood}(p)|\geq 2$$ (2)

Here, *N*_*neighborhood*_ denotes the number of other events in the cylindrical neighborhood of event *p*.

#### Adaptive determination of clustering parameters *ΔR* and *ΔT.*

**Fig 4:**
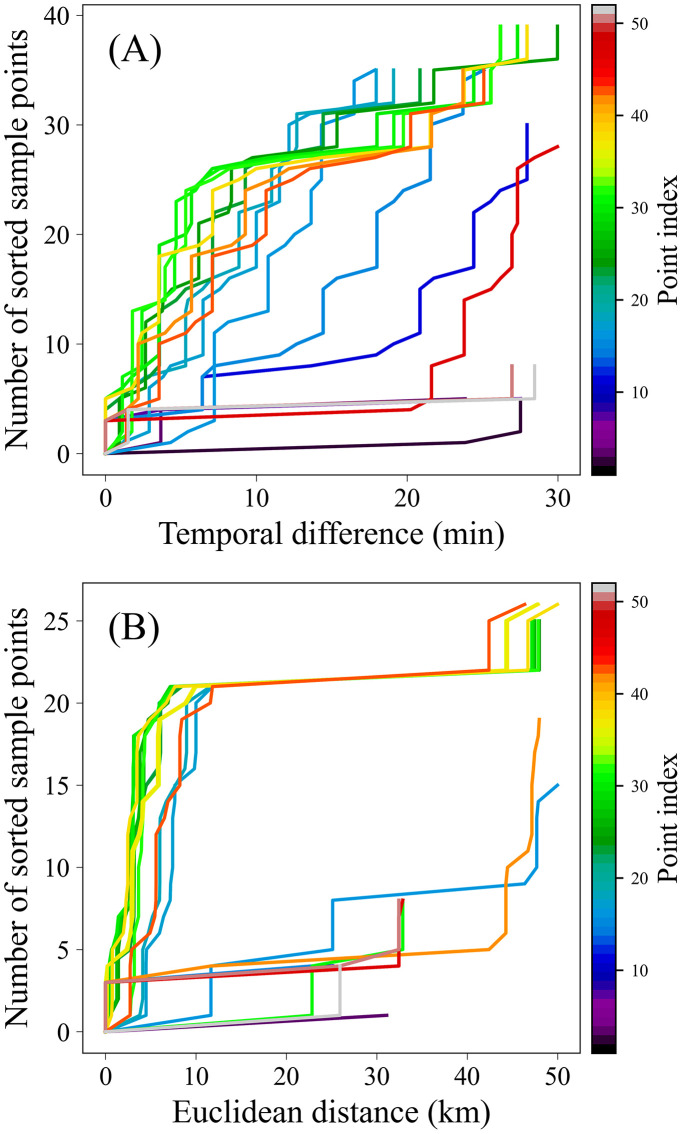
Distribution of (A) temporal difference set (DistT) and (B) spatial distance set (DistS) values by sample number, for the lightning data subset (Fig 3(B)) identified by the CC3D algorithm on 22 March 2015. Note: each curve represents the neighborhood information of a lightning event within a cylindrical region defined by a 50 km radius and a 30-minute temporal window. The horizontal coordinate represents the Euclidean distance or temporal difference between the lightning event and its neighboring points, while the vertical coordinate denotes the number of neighboring points sorted by distance or time difference.

Taking a lightning data subset *D*, identified by the CC3D algorithm on 22 March 2015 (Fig 3B), as an example, the temporal difference list (*DistT*) and spatial distance list (*DistS*) between a given event *p* and other events within a larger cylindrical neighborhood (30-minute temporal window and 50 km spatial radius) are computed. A larger cylindrical neighborhood is defined to avoid including all lightning events in the adaptive algorithm, thereby reducing computational cost. *DistT* or *DistS* is sorted and plotted, with the number of sorted sample points on the y-axis and the corresponding distances or time differences on the x-axis. The neighborhood information of a single lightning event forms an individual curve. Fig 4 presents the sorted distance or time-difference curves for each event within a lightning cluster. The variation in the distribution of the curves is related to the clustering and dispersion characteristics of the lightning events. By examining the shape of each curve, it is found that they closely resemble the receiver of the operating characteristic (ROC) curve [43]. The inflection point near the upper-left corner of the ROC curve represents the model's optimal probability threshold, achieving a high hit rate while maintaining a low false alarm rate. Similarly, the inflection point around 10 km observed in Fig 4B may serve as an optimal distance threshold for these curves, marking a distinct transition in local neighborhood density. This finding aligns with a key assumption of density-based clustering algorithms—that regions of highest local density are typically concentrated within relatively small spatial scales. Evidently, for non-noise events, the inflection point near the upper-left corner can be regarded as the optimal clustering parameter for that event. However, experimental evaluations indicate that the initial cylindrical neighborhood does not ensure that the inflection point on each curve corresponds to the optimal parameter threshold, as ideal clustering results are achieved only when the curve lies closest to the upper-left corner.

**Fig 5:**
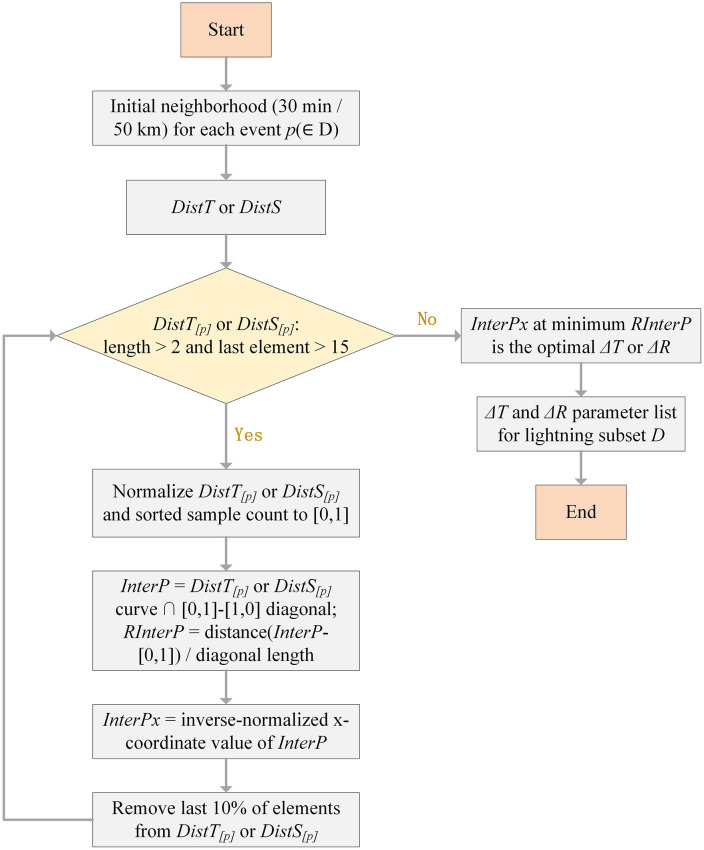
Workflow for determining adaptive clustering parameters.

This study designs a robust algorithmic procedure for obtaining adaptive clustering parameters. The specific steps are as follows (Fig 5):

(1) For each event *p*, the temporal and spatial differences with other events within a larger cylindrical neighborhood (30 min and 50 km) are calculated to construct the *DistT* and *DistS* lists for the lightning data subset *D*.
(2) Each *DistT*_[*p*]_ or *DistS*_[*p*]_ and the corresponding event count are normalized to the [0, 1] range. The intersection point (*InterP*) between the resulting curve and the diagonal line connecting [0, 1] and [1, 0] is then computed. Calculate the ratio *RInterP* of the distance between the *InterP* and the top-left corner point [0,1] to the distance between points [0,1] and [1,0]. The x-coordinate value of *InterP* is back-calculated to the original coordinate space and denoted as *InterPx*.
(3) The last 10% of values in either *DistT*_[*p*]_ or *DistS*_[*p*]_ are iteratively removed, and Step (2) is repeated until the number of elements in *DistT*_[*p*]_ or *DistS*_[*p*]_ is less than 3, and the last value in *DistT*_[*p*]_ or *DistS*_[*p*]_ is less than 15. When *RInterP* is at its minimum, the corresponding *InterPx* is the optimal clustering parameter for event *p*. It is important to note that both the 10% iterative removal rate and the termination threshold of 15 are compromise choices. A removal rate that is too high may result in overly coarse pruning steps, thereby reducing the precision of the *RInterP* parameter. In contrast, an overly low removal rate increases computational time due to insufficient reduction per iteration. Referring to sensitivity analyses in literatures [7,8], the termination threshold used in this study corresponds to the maximum spatial distance and temporal interval. If the threshold is set too low, the spatiotemporal neighborhood may be excessively pruned, which could compromise the accuracy of the resulting *RInterP* value.
(4) By traversing the lightning data subset *D*, we can ultimately obtain an optimal and variable list of spatiotemporal neighborhood thresholds.
(5) Inputting the variable threshold list into the spatio-temporal scan clustering algorithm allows one to obtain the cluster label for each lightning event.

**Fig 6:**
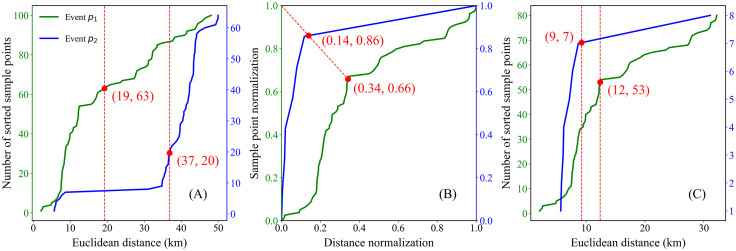
The proposed adaptive clustering algorithm determines the optimal distance parameters for two lightning events, *p*_1_ and *p*_2_. (A) The tangent point determined by the initial cylindrical neighborhood, (B) the tangent point determined after normalization and neighborhood pruning, and (C) the final or optimal tangent point located near the inflection point of the curve.

Table 1 provides a summary of the key parameters used in the CSCAP algorithm. The algorithm was then applied to the lightning data subset identified on March 22, 2015 (Fig 3B). This lightning data subset encompasses multiple convective systems and is highly representative of the overall dataset. Fig 6 illustrates the key process of obtaining the optimal nearest neighbor distance parameter for two lightning events. It can be observed that the tangent points identified by the initial cylinder are significantly higher than the empirical radius distances used in existing research. Interestingly, the adaptively determined radius threshold after neighborhood pruning aligns well with the theoretical situation of lightning clustering.

**Table 1:** Summary of key parameters used in the CSCAP algorithm.

Parameter	Description	Default value	Tuning criteria
*ΔR*	Spatial radius of cylindrical scanning	Adaptive (initial 50 km)	Estimated via *InterPx* from *DistS* curves
*ΔT*	Temporal interval of cylindrical scanning	Adaptive (initial 30 min)	Estimated via *InterPx* from *DistT* curves
*MinPts*	Density threshold for determining core points	2	Common default to suppress false thunderstorms
*RInterP*	Ratio between *InterP*–[0,1] distance and diagonal length	N/A	Measures whether *InterPx* is closest to the top-left corner
*InterPx*	*X*-coordinate of *InterP* mapped back to original scale	N/A	Selected when corresponding *RInterP* reaches its minimum

## Results and discussion

### Coarse-scale identification results of the CC3D algorithm

#### Temporal characteristics of grid clusters.

Table 2 summarizes the monthly number of lightning strokes and the corresponding grid clusters identified by the CC3D algorithm from 2013 to 2015. Substantial variability is evident across both years and months, with lightning activity during the warm-season months far exceeding that in the cold season. In 2013 and 2014, lightning strokes peaked in July and August, with August 2013 alone recording over 2.08 million strokes. In contrast, lightning activity in 2015 was more frequent in the spring, with a peak in April. Similarly, the number of grid clusters identified during the cold season is consistently lower than during the warm season. However, this discrepancy is more pronounced than the ratio of lightning strokes between the two seasons. It is not unexpected that thousands of clusters were identified during the cold-season months despite the low stroke counts, as isolated strokes and scattered noise are assigned the same weight as high-stroke-rate grid cells.

**Table 2:** Monthly counts of lightning strokes and grid clusters and the proportion of grid clusters with more than 500 strokes during 2013–2015.

Year	Jan	Feb	Mar	Apr	May	Jun	Jul	Aug	Sep	Oct	Nov	Dec
Monthly number of lightning strokes												
2013	2787	13592	353372	282499	397960	785690	1163612	2089767	180207	1918	3346	3313
2014	3325	2269	210323	134609	156706	208443	1224607	605719	286096	12398	29493	2541
2015	2703	77056	82059	883683	729301	550164	485283	538656	89852	27591	36420	8466
Monthly number of grid clusters												
2013	1637	2231	4781	6017	10370	9163	8494	8129	5518	1399	2140	1836
2014	1458	683	3563	3874	5993	7207	7154	6046	3599	3696	3781	1952
2015	1435	1430	2035	3136	6702	7846	6207	6267	5675	2519	2427	1212
Number or proportion of grid clusters with more than 500 lightning strokes												
2013	0 (0%)	5 (0.2%)	14 (0.3%)	12 (0.2%)	20 (0.2%)	18 (0.2%)	32 (0.4%)	31 (0.4%)	7 (0.1%)	0 (0%)	0 (0%)	0 (0%)
2014	0 (0%)	0 (0%)	8 (0.2%)	11 (0.3%)	13 (0.2%)	17 (0.2%)	34 (0.5%)	32 (0.5%)	17 (0.5%)	6 (0.2%)	4 (0.1%)	0 (0%)
2015	0 (0%)	11 (0.8%)	12 (0.6%)	16 (0.5%)	15 (0.2%)	28 (0.4%)	31 (0.5%)	44 (0.7%)	6 (0.1%)	11 (0.4%)	14 (0.6%)	3 (0.2%)

Since thunderstorms with high stroke rates often exert greater atmospheric influence, the proportion of grid clusters with more than 500 lightning strokes was further analyzed (Table 2). The selected lightning threshold is representative. Although the number of high-flash-rate clusters decreases with increasing threshold, it tends to stabilize when the threshold reaches 500 strokes, indicating a robust cutoff for identifying intense thunderstorm activity. A total of 472 such high-stroke clusters were identified over the three years. Nonetheless, these intense clusters represented less than 1% of the total clusters in any given month, indicating that most clusters were associated with only a small number of lightning strokes. This also helps explain why a relatively large number of clusters can be identified during winter months, even when lightning activity is minimal.

#### Spatial characteristics of grid clusters.

**Fig 7:**
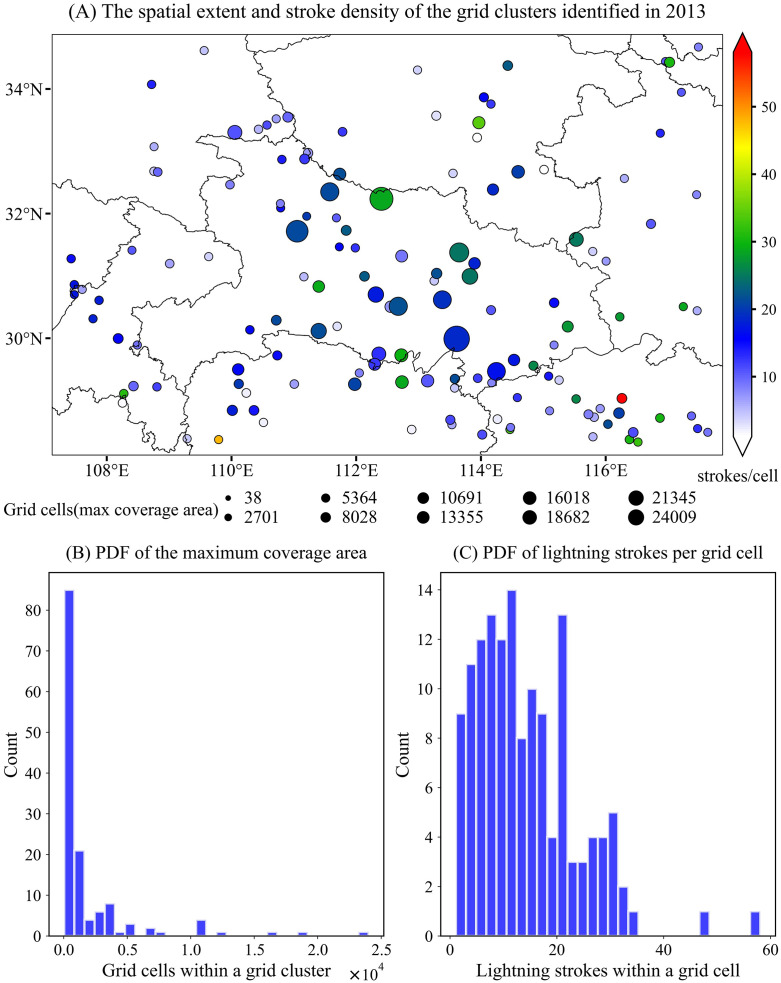
Statistical properties of high-flash-rate (≥500 lightning strokes) grid clusters identified in 2013 within the study region: (A) Spatial distribution of cluster centroids; (B, C) Probability distribution functions (PDFs) of the number of grids per cluster and the number of lightning strokes per grid, respectively. Note: the size of each circle represents the total number of spatiotemporal grids within the cluster, while the color indicates the number of lightning strokes per grid.

**Fig 8:**
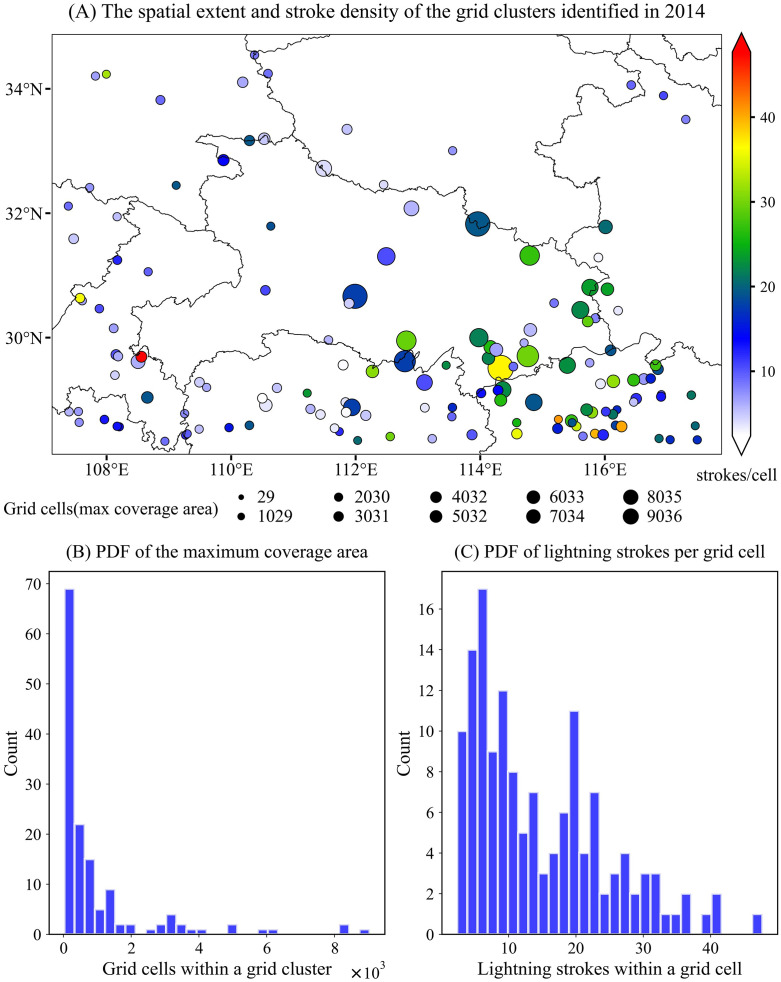
Similar to Fig 7, but for the year 2014.

**Fig 9:**
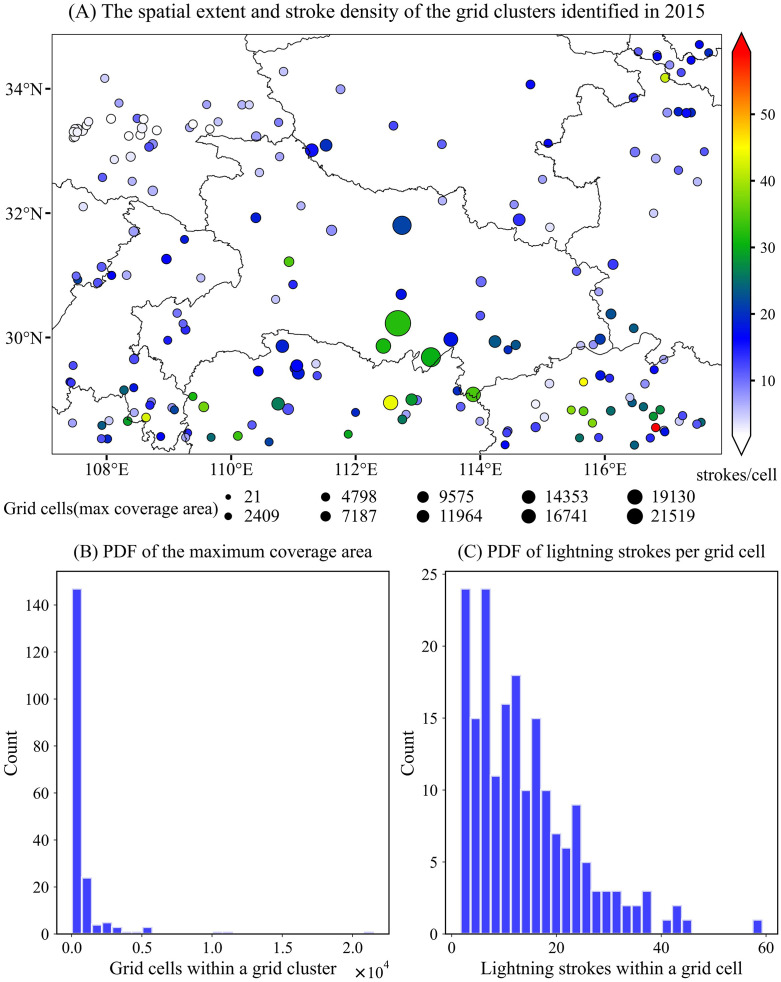
Similar to Fig 7, but for the year 2015.

Figs 7–9 show the spatial distributions of 139, 142, and 191 high-flash-rate (≥500 lightning strokes) grid clusters identified in 2013, 2014, and 2015, respectively. Panel (1) in each figure illustrates the geographic locations of the centroids of these coarse-scale, spatiotemporally continuous clusters. The maximum area influenced by lightning strokes and the cluster intensity are indicated by the size and color of the circles, respectively. In 2013, grid clusters with extensive spatial coverage were primarily concentrated in the central part of the study region (Hubei Province), with the largest cluster consisting of more than 24,000 spatiotemporal grids (Fig 7). Although this cluster may comprise multiple deep convective systems, these storms were spatiotemporally connected or adjacent—reflecting the merging, propagation, or evolution of convective storms that expand their influence area. The cluster with the highest intensity was located in the southeastern part of the study area (northern Jiangxi Province), where an average of 58 lightning strokes occurred per grid cell.

In 2014, high–stroke-rate grid clusters occurred frequently in the southern part of the study region, particularly over the middle and lower reaches of the Yangtze River, where numerous clusters with large areal extents and high intensities were identified (Fig 8). Interestingly, these clusters were situated near hydrologically significant features including Dongting Lake in northeastern Hunan, the Yangtze River in southeastern Hubei, and Poyang Lake in northern Jiangxi. An ample moisture supply is a necessary condition for the initiation and sustained development of convective systems. The cluster with the highest stroke intensity was located along the western border of Chongqing Municipality, which may be related to local topographic effects. Warm, moist air masses originating from the southeast or southwest can be blocked and lifted by the mountainous terrain to the north, triggering convection and enhancing vertical motion through moisture convergence. Previous studies have shown that the frequency of lightning within thunderstorms is closely associated with the velocity and volume of updrafts.

In 2015, high–stroke-rate grid clusters were distributed across the entire study region (Fig 9). Although the total number of lightning strokes was considerably lower than in 2013 but higher than in 2014, the number of high–stroke-rate clusters was approximately 1.3 times that of both 2013 and 2014. Compared to the previous two years, more clusters were observed in the northeastern and northwestern parts of the region in 2015; however, their maximum areal extents and intensities were generally weaker than those in the central and southern portions of the study area. The enhanced upper-level westerly jet stream may have contributed to the increased lightning activity in the northern region. As shown in Table 2, the number of grid clusters during the cold-season months was significantly higher than in 2013 and 2014. Previous studies have suggested that the upper-level jet is strongest during the cold season and that the interaction between cold advection aloft and warm, moist air in the mid-to-lower troposphere is a primary mechanism for convective initiation. Other characteristics resembled those in Fig 7, such as the widespread lightning activity in Hubei Province and the occurrence of high-intensity clusters in northern Jiangxi.

### Fine-scale identification results of the CSCAP algorithm

#### Rationality evaluation of clustering results.

Subsets of lightning data identified by the CC3D algorithm were used as input for the CSCAP density-based clustering algorithm to obtain all lightning strokes belonging to the same convective cloud. The inherent physical properties of convective clouds (coverage area and duration) imply that adjacent lightning strokes within a thunderstorm are spatiotemporally correlated; therefore, a spatiotemporal neighborhood-based clustering algorithm is the optimal choice. The key to this algorithm lies in determining the temporal and spatial clustering parameters, which refer to the maximum allowable distance and time interval between adjacent lightning strokes. Changes in these clustering parameters also affect the physical properties of the resulting clusters. Table 3 presents the identification results of thunderstorms in the study area during 2013–2015 using the adaptive-parameter spatiotemporal clustering method (CSCAP) and the fixed-parameter spatiotemporal clustering method (CSCGP). It is noteworthy that CSCGP is equivalent to the standard ST-DBSCAN algorithm, whereas CSCAP extends ST-DBSCAN by incorporating an adaptive mechanism for spatiotemporal parameter selection. Although the Table 3 does not include statistics on thunderstorm cloud coverage, the changes in cluster numbers are sufficient to reflect variations in coverage area. Specifically, as the distance threshold increases, adjacent convective cells merge into a single cluster, resulting in fewer clusters but larger coverage areas.

**Table 3:** Fine-scale identification results of lightning clusters in the study area during 2013–2015 using adaptive-parameter spatiotemporal clustering (CSCAP) and fixed-parameter spatiotemporal clustering (CSCGP) methods.

Method	Spatial distance (km)	Time interval (min)	Number of clusters	Ratio of lightning strokes involved in clustering
CSCAP	–	–	771033	98.988% (11547713/11665826)
CSCGP	5	10	775484	98.148% (11449734/11665826)
CSCGP	5	15	774100	98.250% (11461688/11665826)
CSCGP	5	20	773525	96.319% (11469687/11665826)
CSCGP	10	10	771858	98.612% (11503908/11665826)
CSCGP	10	15	769688	98.722% (11516679/11665826)
CSCGP	10	20	768665	98.796% (11525325/11665826)
CSCGP	15	10	769984	98.861% (11532934/11665826)
CSCGP	15	15	767543	98.971% (11545766/11665826)
CSCGP	15	20	766383	99.047% (11554672/11665826)
CSCGP	20	10	768975	99.030% (11552620/11665826)
CSCGP	20	15	766606	99.141% (11565574/11665826)
CSCGP	20	20	765729	99.220% (11574791/11665826)

To prevent individual convective cells from being merged into the same cluster while retaining most lightning strokes, a balance must be achieved between the number of clusters and the proportion of strokes included in clustering. Different parameter combinations were set using transitional thresholds of 5 km and 5 minutes, with the statistical characteristics of the clustering results summarized in Table 3. The spatiotemporal thresholds of 5 km and 10 minutes identified the most significant number of thunderstorms, totaling 775,484 clusters (mostly single-stroke clusters). However, the minimum proportion of usable strokes was only 98.148%, indicating that a substantial number of strokes were excluded from clustering due to not meeting the thresholds, and some larger thunderstorm systems were fragmented into multiple convective systems. As the spatial or temporal thresholds increased, the number of lightning clusters decreased while the proportion of clustered strokes increased.

For thresholds of 20 km and 20 minutes, 99.220% of lightning strokes were included in the clustering, but only 765,729 clusters were identified. This suggests that more relaxed thresholds incorporate more isolated events or noise into clustering, thereby increasing the usable stroke proportion. However, adjacent convective systems tend to merge, reducing the cluster counts. Therefore, the optimal clustering algorithm should maximize the number of identified clusters while retaining as many valid strokes as possible. The CSCAP algorithm identified 771,033 thunderstorms with an effective stroke proportion of 98.988%, well satisfying these criteria.

#### Two cases of clustering results.

**Fig 10:**
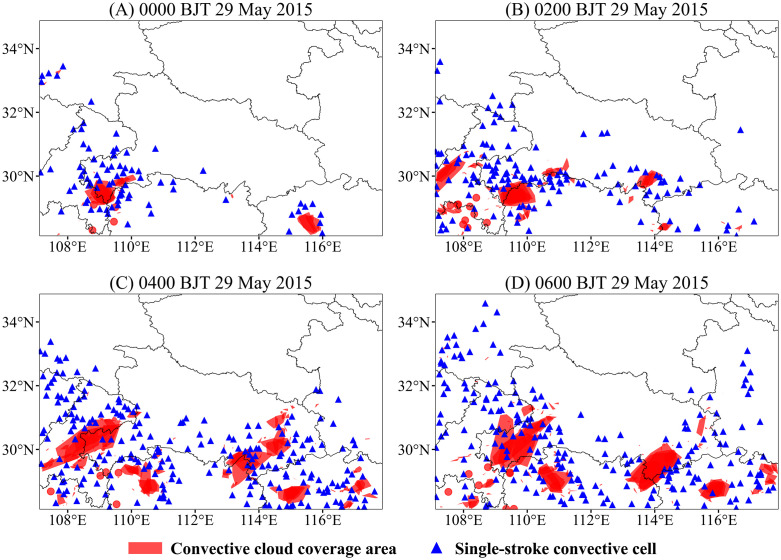
Thunderstorm clouds were identified and extracted by the CSCAP algorithm from 0000–0600 BJT (Beijing Time) on 29 May 2015. The red polygons represent the boundaries extracted using the convex hull algorithm based on clustered strokes within an hour. At the same time, the blue triangles indicate weak convective cells associated with only a single lightning stroke.

**Fig 11:**
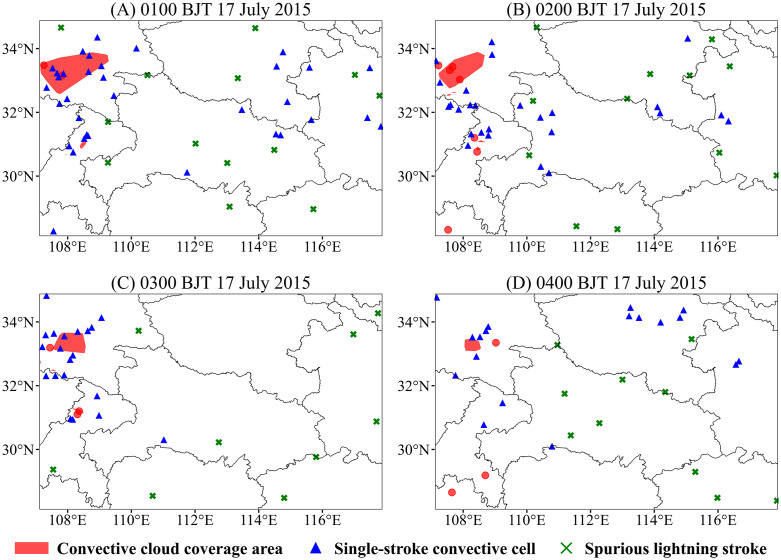
Similar to Fig 10, but for 0100–0400 BJT (Beijing Time) on 17 July 2015. In addition, green crosses indicate isolated noise points that have no associated neighbors under either the 26-grid connected neighborhood and the spatiotemporal scanning algorithm.

Figs 10 and 11 present two specific cases identified by the fine-scale clustering algorithm, derived from lightning data subsets comprising tens of thousands and several hundred strokes, respectively. The convective cloud coverage corresponds to the outer boundary of the identified lightning clusters. Single-flash convective cells are defined as strokes that are not isolated at the coarse spatiotemporal scale but fail to meet the threshold criteria of the CSCAP algorithm and are thus excluded from fine-scale clustering. Noise flashes are those identified as isolated events in both coarse- and fine-scale clustering.

Fig 10 effectively visualizes the spatiotemporal evolution of a long-lived thunderstorm system, capturing not only its initiation, development, and dissipation but also dynamic processes such as propagation, movement, splitting, and merging. This provides insight into the complex behavior of deep moist convection in the region. At 0000 BJT (Beijing Time) on 29 May 2015, two thunderstorm clusters were observed in the study area: one over the southwestern corner of Hubei Province (Enshi region) and a smaller one in northern Jiangxi Province. By 0200 BJT, the Enshi thunderstorm had moved eastward into southwestern Hunan and developed a narrow, elongated cell in its northeastern flank; the northern Jiangxi storm dissipated, while new clusters emerged along the northwestern border of Jiangxi, southeastern Hubei, and western Chongqing. At 0400 BJT, the Chongqing thunderstorm propagated eastward with a notable increase in areal extent; the Enshi-originated storm moved fully into Hunan; meanwhile, the Hubei storm expanded northward, and several smaller convective cells were triggered in eastern Jiangxi. By 0600 BJT, the Chongqing thunderstorm fully overlaid the Enshi area; the southeastern Hubei cluster shrank due to dissipation; and the east Jiangxi storm cells gradually merged while shifting eastward.

On 17 July 2015, a large-area thunderstorm occurred in the central-southern region of Shaanxi Province, lasting up to four hours and revealing a temporal pattern in the evolution of the convective cloud coverage (Fig 11). At 0100 BJT, the convective storm was in its early to mature stage, exhibiting the most extensive coverage area. This expansion was attributed to abundant moisture supply and strong updrafts that supported the vertical development and horizontal spreading of the cloud system. In the subsequent hours, the storm transitioned into a dissipating stage, and the coverage area showed a decreasing trend. This contraction was primarily driven by weakening updrafts and increasing precipitation. The updrafts consumed much of the convective energy, while the downdrafts disrupted the inflow of moisture. Without a sustained moisture supply, the storm coverage reached its minimum by 0400 BJT, indicating a tendency toward the dissipation of deep convective clouds.

Overall, the spatiotemporal evolution and dynamic processes of the convective system were closely linked to the surrounding environmental conditions. The initiation and dissipation of convection were influenced by atmospheric instability, moisture availability, and the prevailing synoptic-scale systems. The complex interactions among updrafts, downdrafts, and atmospheric background conditions governed the movement, splitting, and merging of storm cells.

## Conclusions

This study presents the CC3D-CSCAP method for extracting lightning clusters from large-scale cloud-to-ground lightning datasets. CC3D partitions the dataset into spatiotemporally independent subsets, reducing the computational cost of density-based clustering. CSCAP adaptively determines clustering parameters from the spatial and temporal proximities of neighboring lightning strokes, accommodating variations in lightning density across thunderstorm systems.

Coarse-scale grid clusters over eastern and central China were identified from the 2013–2015 lightning dataset using the CC3D algorithm. Their spatial distribution appeared to be associated with terrain, moisture transport pathways, and upper-level jet streams. Among them, high-flash-rate clusters (>500 strokes) accounted for less than 1% of all grid clusters. By applying the CSCAP algorithm to lightning subsets extracted by the CC3D algorithm, fine-scale lightning clusters were identified with a lightning utilization rate of 98.988%, yielding 771,033 convective systems. Case analyses suggest that CC3D-CSCAP can reliably capture key stages in the thunderstorm life cycle, including initiation, propagation, splitting, merging, and dissipation.

The methodology presented in this study provides valuable capabilities for thunderstorm nowcasting, convective climatology investigations, and assessments of global electrical activity. However, its application is currently limited by the availability of lightning datasets spanning larger spatial domains and longer temporal periods, thereby restricting robust evaluation of the method's confidence. Importantly, the proposed framework is adaptable to other spatiotemporally structured datasets, indicating broad potential beyond lightning stroke analysis.

## Supporting information

S1 Fig Hierarchical relationships among key terms used in this study. (TIF)

S2 Data Sample lightning stroke data. (CSV)

## References

[1] Byers HR, Braham RR Jr. The Thunderstorm. U.S. Government Printing Office; 1949. p. 287.

[2] Brooks HE. Severe thunderstorms and climate change. Atmos Res. 2013;123:129–38. doi: 10.1016/j.atmosres.2012.04.002

[3] Elkhouly M, Zick SE, Ferreira MAR. Long term temporal trends in synoptic-scale weather conditions favoring significant tornado occurrence over the central United States. PLoS One. 2023;18(2):e0281312. doi: 10.1371/journal.pone.0281312 36812264 PMC9946245

[4] Terti G, Ruin I, Anquetin S, Gourley JJ. A Situation-Based Analysis of Flash Flood Fatalities in the United States. Bull Am Meteorol Soc. 2017;98(2):333–45. doi: 10.1175/bams-d-15-00276.1

[5] Shi M, Zhang W, Fan P, Chen Q, Liu Z, Li Q, et al. Modelling deep convective activity using lightning clusters and machine learning. Int J Climatol. 2022;42(2):952–73. doi: 10.1002/joc.7282

[6] Taszarek M, Allen J, Púčik T, Groenemeijer P, Czernecki B, Kolendowicz L, et al. A Climatology of Thunderstorms across Europe from a Synthesis of Multiple Data Sources. J Clim. 2019;32(6):1813–37. doi: 10.1175/jcli-d-18-0372.1

[7] Galanaki E, Lagouvardos K, Kotroni V, Flaounas E, Argiriou A. Thunderstorm climatology in the Mediterranean using cloud-to-ground lightning observations. Atmos Res. 2018;207:136–44. doi: 10.1016/j.atmosres.2018.03.004

[8] Hutchins ML, Holzworth RH, Brundell JB. Diurnal variation of the global electric circuit from clustered thunderstorms. JGR Space Physics. 2014;119(1):620–9. doi: 10.1002/2013ja019593

[9] Fan P, Zheng D, Zhang Y, Gu S, Zhang W, Yao W, et al. A Performance Evaluation of the World Wide Lightning Location Network (WWLLN) over the Tibetan Plateau. J Atmos Oceanic Technol. 2018;35(4):927–39. doi: 10.1175/jtech-d-17-0144.1

[10] Zheng D, Zhang Y, Meng Q, Chen L, Dan J. Climatological Comparison of Small- and Large-Current Cloud-to-Ground Lightning Flashes over Southern China. J Clim. 2016;29(8):2831–48. doi: 10.1175/jcli-d-15-0386.1

[11] Zheng D, Zhang Y, Meng Q, Chen L, Dan J. Climatology of lightning activity in South China and its relationships to precipitation and convective available potential energy. Adv Atmos Sci. 2016;33(3):365–76. doi: 10.1007/s00376-015-5124-5

[12] Yuan S, Qie X, Jiang R, Wang D, Sun Z, Srivastava A, et al. Origin of an Uncommon Multiple‐Stroke Positive Cloud‐to‐Ground Lightning Flash With Different Terminations. JGR Atmospheres. 2020;125(15):e2019jd032098. doi: 10.1029/2019jd032098

[13] Dennis AS. The Flashing Behavior of Thunderstorms. J Atmos Sci. 1970;27(1):170–2. doi: 10.1175/1520-0469(1970)027<0170:tfbot>2.0.co;2

[14] Yair YY, Aviv R, Ravid G. Clustering and synchronization of lightning flashes in adjacent thunderstorm cells from lightning location networks data. J Geophys Res. 2009;114(D9). doi: 10.1029/2008jd010738

[15] Harel M, Price C. Thunderstorm Trends over Africa. J Clim. 2020;33(7):2741–55. doi: 10.1175/jcli-d-18-0781.1

[16] Hayward L, Whitworth M, Pepin N, Dorling S. Thunderstorm tracking in Northwest Europe for enhanced hazard preparedness. Int J Climatol. 2023;43(11):4894–916. doi: 10.1002/joc.8123

[17] Srivastava A, Liu D, Xu C, Yuan S, Wang D, Babalola O, et al. Lightning Nowcasting with an Algorithm of Thunderstorm Tracking Based on Lightning Location Data over the Beijing Area. Adv Atmos Sci. 2022;39(1):178–88. doi: 10.1007/s00376-021-0398-2

[18] Strauss C, Rosa MB, Stephany S. Spatio-temporal clustering and density estimation of lightning data for the tracking of convective events. Atmos Res. 2013;134:87–99. doi: 10.1016/j.atmosres.2013.07.008

[19] Zscheischler J, Mahecha MD, Harmeling S, Reichstein M. Detection and attribution of large spatiotemporal extreme events in Earth observation data. Ecol Inform. 2013;15:66–73.

[20] Vogel MM, Zscheischler J, Fischer EM, Seneviratne SI. Development of Future Heatwaves for Different Hazard Thresholds. J Geophys Res Atmos. 2020;125(9):e2019JD032070. doi: 10.1029/2019JD032070 32728502 PMC7380308

[21] Guan Y, Gu X, Slater LJ, Li L, Kong D, Liu J, et al. Tracing anomalies in moisture recycling and transport to two record-breaking droughts over the Mid-to-Lower Reaches of the Yangtze River. J Hydrol (Amst). 2022;609:127787.

[22] Huang Y, Fan Y, Cai L, Cheng S, Wang J. A New Thunderstorm Identification Algorithm Based on Total Lightning Activity. Earth Space Sci. 2022;9(4):e2021ea002079. doi: 10.1029/2021ea002079

[23] Mezuman K, Price C, Galanti E. On the spatial and temporal distribution of global thunderstorm cells. Environ Res Lett. 2014;9(12):124023. doi: 10.1088/1748-9326/9/12/124023

[24] Ester M, Kriegel H, Sander J, Xu X. A density-based algorithm for discovering clusters in large spatial databases with noise. 1996. p. 226–31.

[25] Rodriguez A, Laio A. Machine learning. Clustering by fast search and find of density peaks. Science. 2014;344(6191):1492–6. doi: 10.1126/science.1242072 24970081

[26] Wang Y, Pang W, Zhou Y. Density propagation based adaptive multi-density clustering algorithm. PLoS One. 2018;13(7):e0198948. doi: 10.1371/journal.pone.0198948 30020928 PMC6051564

[27] Okada M, Nagata K, Watanabe N, Tada M. Computational Learning Analytics to Estimate Location-Based Self-Regulation Process of Real-World Experiences. IEEE Trans Learning Technol. 2024;17:445–61. doi: 10.1109/tlt.2023.3262598

[28] Li H, Jia P, Wang X, Yang Z, Wang J, Kuang H. Ship carbon dioxide emission estimation in coastal domestic emission control areas using high spatial-temporal resolution data: A China case. Ocean Coast Manag. 2023;232:106419. doi: 10.1016/j.ocecoaman.2022.106419

[29] Bao L, Liu Z, Miao R, Chen Z, Zhang B, Guo P, et al. Spatiotemporal clustering analysis of shared electric vehicles based on trajectory data for sustainable urban governance. J Clean Prod. 2023;412:137373.

[30] Cai L, Li Y, Chen M, Zou Z. Tropical cyclone risk assessment for China at the provincial level based on clustering analysis. Geomat Nat Hazard Risk. 2020;11(1):869–86.

[31] Cheng S, Wang J, Cai L, Zhou M, Su R, Huang Y, et al. Characterising the dynamic movement of thunderstorms using very low- and low-frequency (VLF/LF) total lightning data over the Pearl River Delta region. Atmos Chem Phys. 2022;22(15):10045–59. doi: 10.5194/acp-22-10045-2022

[32] Yaohui L, Zhengming M, Fang Y. Adaptive density peak clustering based on K-nearest neighbors with aggregating strategy. Knowl Based Syst. 2017;133:208–20. doi: 10.1016/j.knosys.2017.07.010

[33] Shi M, Liu X, Fan P, Zhang W, Gao W. Evaluation and application analysis of kilometer-scale convective parameters derived from a statistical downscaling method over Central China. Clim Dyn. 2023;61(9):4563–86.

[34] Shi M, Fan P, Pan X, Gao W, Zhang W, Liang D, et al. The relationship between the land features and thermodynamic parameters and the thunderstorm hours over central and eastern China. Weather Forecast. 2025;40(5):719–39.

[35] Chen M, Wang Y, Gao F, Xiao X. Diurnal variations in convective storm activity over contiguous North China during the warm season based on radar mosaic climatology. J Geophys Res. 2012;117(D20). doi: 10.1029/2012jd018158

[36] Fan P, Zheng D, Zhang Y, Gu S, Zhang W, Yao W, et al. A performance evaluation of the world wide lightning location network (WWLLN) over the Tibetan Plateau. J Atmos Ocean Technol. 2018;35(4):927–39.

[37] Cummins KL, Murphy MJ, Bardo EA, Hiscox WL, Pyle RB, Pifer AE. A combined TOA/MDF technology upgrade of the US National Lightning Detection Network. J Geophys Res Atmos. 1998;103(D8):9035–44.

[38] Rosenfeld A, Pfaltz JL. Sequential Operations in Digital Picture Processing. J ACM. 1966;13(4):471–94. doi: 10.1145/321356.321357

[39] Wu K, Otoo E, Suzuki K. Two strategies to speed up connected component labeling algorithms. Berkeley, CA (United States): Lawrence Berkeley National Lab (LBNL); 2005.

[40] Luo M, Lau N, Liu Z, Wu S, Wang X. An Observational Investigation of Spatiotemporally Contiguous Heatwaves in China From a 3D Perspective. Geophys Res Lett. 2022;49(6):e2022gl097714. doi: 10.1029/2022gl097714

[41] Reddy PJ, Perkins-Kirkpatrick SE, Sharples JJ. Interactive influence of ENSO and IOD on contiguous heatwaves in Australia. Environ Res Lett. 2021;17(1):014004. doi: 10.1088/1748-9326/ac3e9a

[42] Birant D, Kut A. ST-DBSCAN: An algorithm for clustering spatial–temporal data. Data Knowl Eng. 2007;60(1):208–21. doi: 10.1016/j.datak.2006.01.013

[43] Bradley AP. The use of the area under the ROC curve in the evaluation of machine learning algorithms. Pattern Recognit. 1997;30(7):1145–59.

