# Supplementary figures and images for: A lightning cluster identification method considering multi-scale spatiotemporal neighborhood relationships
Source: PLoS One. 2025 Oct 3;20(10):e0333207. doi: 10.1371/journal.pone.0333207 (PMC12494261; doi:10.1371/journal.pone.0333207)

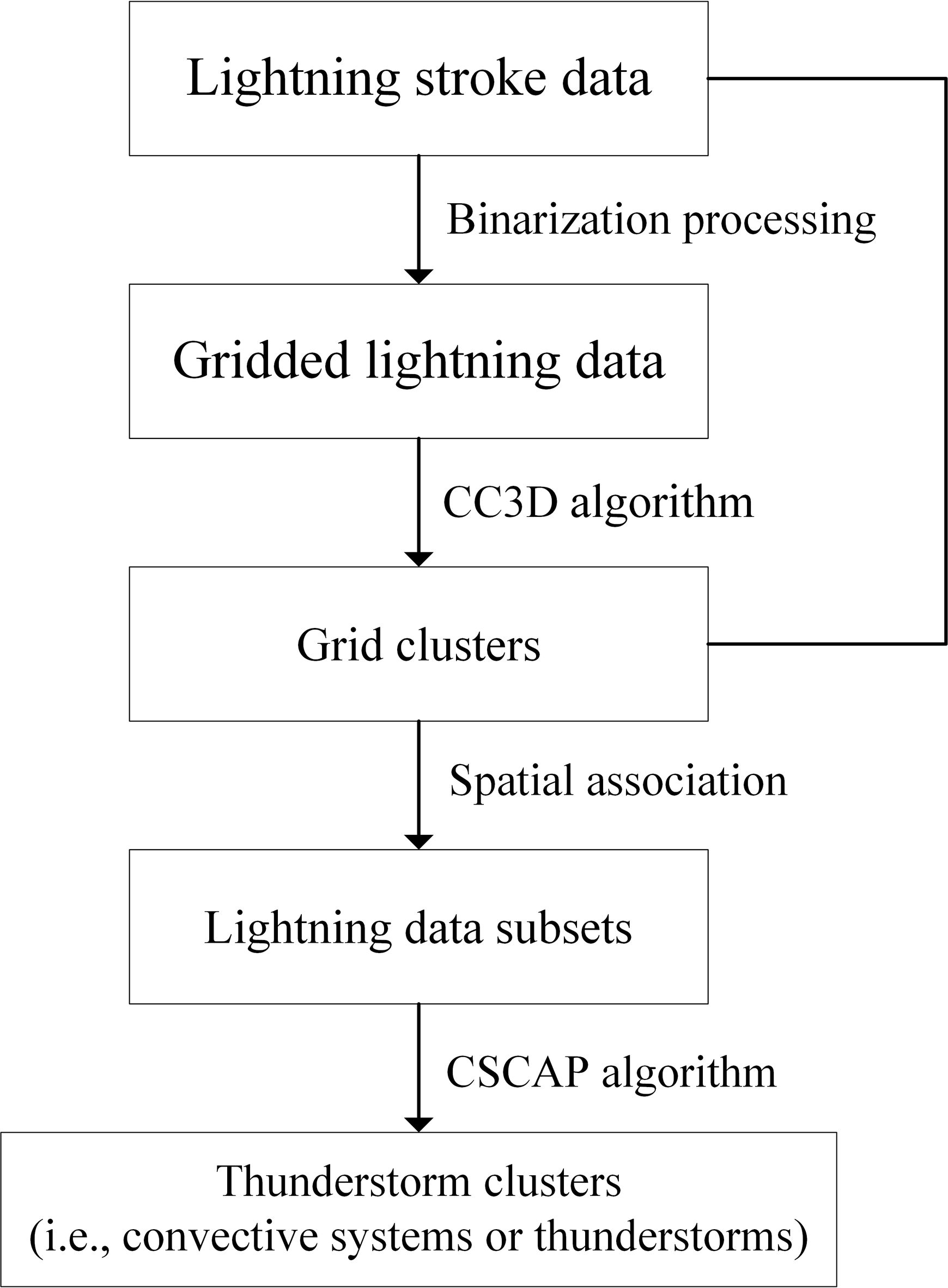

Supplement: S1 Fig — (TIF) [file pone.0333207.s001.tif]
